# Gene Therapy for Osteoarthritis: Viral and Non-Viral Delivery Strategies

**DOI:** 10.3390/v18101151

**Published:** 2026-10-17

**Authors:** Mingcai Zhang, Joshua Mohess, Joseph Cheung, Quanhan Du, Jianming Qiu, Jinxi Wang

**Affiliations:** 1 Harrington Laboratory for Molecular Orthopedics, Department of Orthopedic Surgery, University of Kansas Medical Center, Kansas City, KS 66160, USA; mzhang@kumc.edu (M.Z.); jmohess@kumc.edu (J.M.); jcheung@kumc.edu (J.C.); 2 Department of Microbiology, Molecular Genetics and Immunology, University of Kansas Medical Center, Kansas City, KS 66160, USA; qpd5031@psu.edu (Q.D.); jqiu@kumc.edu (J.Q.); 3 Department of Biochemistry and Molecular Biology, University of Kansas Medical Center, Kansas City, KS 66160, USA

**Keywords:** osteoarthritis, gene therapy, viral vectors, non-viral delivery, clinical trials

## Abstract

Osteoarthritis (OA) is the most prevalent joint disease, which is pathologically characterized by articular cartilage degradation, chronic synovial inflammation, subchondral bone remodeling, and osteophyte formation. Patients with OA suffer from joint pain, deformity, and functional impairment. Current therapies provide symptomatic relief but fail to halt or reverse disease progression. Gene therapy is being investigated for cartilage repair and modulation of intra-articular inflammation, with the aim of modifying disease progression. This narrative review summarizes the recent advances in viral and non-viral gene therapy approaches for OA, including therapeutic gene targets in preclinical and clinical trials. Potentially therapeutic genes can be injected into the joint cavity and transferred into specific joint tissue cells through viral vectors or non-viral carriers depending on the nature of transgenes, delivery materials, and targeting cells. Gene delivery to articular chondrocytes remains challenging as the cells are positioned in a dense extracellular matrix, while synovial fluid may further contribute to degradation or inactivation of delivered agents. Viral vectors, particularly adeno-associated virus (AAV), can support sustained transgene expression in joint tissues, although cartilage penetration efficiency remains to be improved. Non-viral carriers can deliver nucleic acids to synovial cells and, with suitable formulations, articular chondrocytes. Viral and non-viral delivery approaches may offer complementary benefits; integrating the advantages of both viral and non-viral platforms could potentially improve the efficacy and safety of gene therapy for OA. Finally, strategies to overcome translational barriers and future directions towards clinical applications are discussed.

## 1. Introduction

Osteoarthritis (OA) is the most common form of arthritis, affecting over 300 million people worldwide. Patients with OA suffer from chronic joint pain and functional impairment, leading to significant socioeconomic burden [1]. The disease is pathologically characterized by progressive articular cartilage degradation, subchondral bone remodeling, chronic synovial inflammation, and osteophyte formation [2]. Current therapies, including non-steroidal anti-inflammatory drugs (NSAIDs), corticosteroid injections, and joint fusion or replacement, provide symptomatic relief but do not modify disease progression [1,3].

Gene therapy introduces or modifies genetic material to achieve a therapeutic effect [4]. In OA, gene supplementation commonly aims to produce protective proteins locally. Gene editing alters genomic sequences, whereas gene silencing reduces expression without necessarily changing DNA. This review also discusses transient mRNA and siRNA delivery as related nucleic-acid approaches; delivery alone is not evidence of editing or therapeutic benefit. The research field of gene therapy has advanced rapidly in the last two decades, from experimental testing in rare diseases to modern techniques that can edit, insert, and regulate genes associated with a variety of diseases. Potentially therapeutic genes can be transferred into specific cells through viral vectors including adeno-associated virus (AAV), adenovirus (Ad), and lentivirus, or non-viral carriers such as nanoparticles, exosomes, and physical methods, depending on the nature of transgenes, diseases, and targeting cells. After rigorous clinical trials, the U.S. Food and Drug Administration (FDA) has approved the use of gene therapy for several diseases such as inherited retinal dystrophy (Luxturna), spinal muscular atrophy (Zolgensma), hemophilia A and B (Roctavian; Hemgenix), and β-thalassemia and sickle cell disease (Zynteglo and Casgevy) [5,6,7].

Gene therapy offers a promising alternative for treatment of OA through intra-articular gene delivery of a transgene into a relatively confined joint cavity, as OA primarily affects a limited number of load-bearing joints. Intra-articular gene transfer provides direct delivery of therapeutic genetic molecules to joint tissues for long-term disease modulation to inhibit catabolism and/or promote anabolic repair, with limited systemic exposure [8]. Advances in viral and non-viral delivery systems, genome editing tools, and cartilage-targeted delivery methods have expanded the potential for OA gene therapy, with multiple preclinical studies demonstrating efficacy in cartilage repair and inflammation suppression [8,9]. Self-complementary (sc)-rAAV2.5-IL-1Ra (now known as GNSC-001) is a scAAV2.5 vector designed for intra-articular gene therapy, expressing the interleukin-1 receptor antagonist protein (IL-1Ra), encoded by *IL1RN*. The published phase 1 study (NCT02790723) reported sustained local IL-1Ra expression and acceptable tolerability in nine patients; pain improvements were exploratory because the study lacked a placebo group [10]. GNSC-001 is being evaluated in a separate phase 1b study (NCT05835895). PCRX-201 (formerly FX201) is a high-capacity adenoviral vector designed to express IL-1Ra under an inflammation-inducible promoter for knee OA (NCT04119687; phase 1). *IL1RN*, IL-1Ra, and IL-1R denote the gene, antagonist protein, and receptor, respectively.

### Literature Identification and Scope

This is a narrative review focused on osteoarthritis gene therapy using viral vectors (AAV, Ad, and lentivirus) and non-viral approaches, e.g., mRNA and exosome from publications available in PubMed through September 2026. Studies were selected for relevance to joint delivery, OA target biology, animal proof of concept, or human safety and efficacy. ClinicalTrials.gov records for the selected clinical programs were checked on the same date. Table 1 compares the difference between viral and non-viral gene-delivery approaches for OA, Table 2 summarizes target-biology evidence, Table 3 illustrates selected *in vivo* delivery in animal studies, and Table 4 summarizes selected knee-OA clinical trials.

## 2. Normal Anatomical and Physiological Basis of the Joint

**Figure 1:**
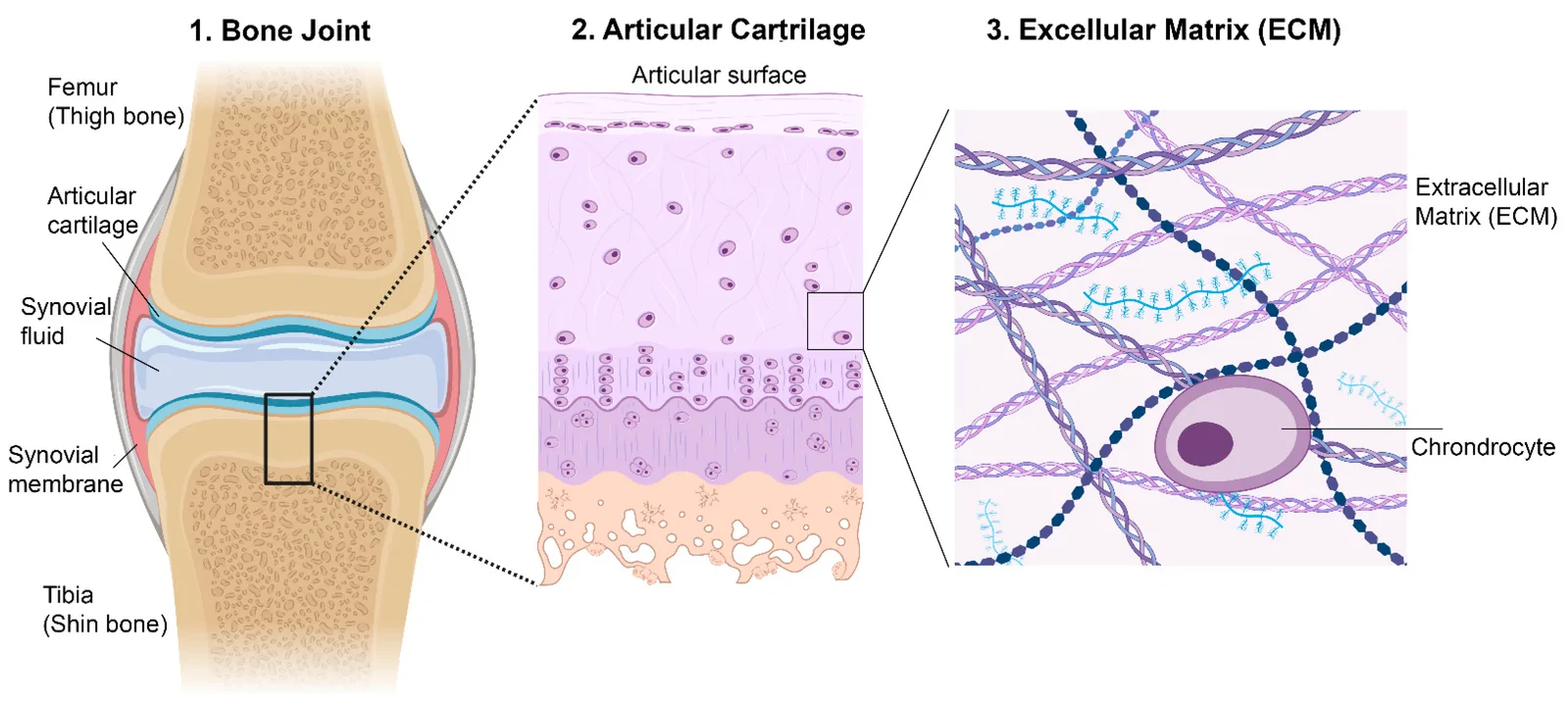
Joint structure and articular cartilage components. Schematic of a synovial joint and the organization of articular cartilage. Left (**1**): Joint structure showing bone ends covered by cartilage and surrounded by synovial fluid and membrane. Middle (**2**): Cartilage with chondrocytes embedded in the extracellular matrix (ECM). Right (**3**): ECM composition, mainly collagen type II and proteoglycans, which provide strength and resistance to compression. This structure supports smooth joint movement and load distribution.

Articular cartilage is a highly specialized, avascular connective tissue that covers the ends of articulating bones and enables smooth, low-friction joint movement. It is composed of sparsely distributed chondrocytes embedded within a dense extracellular matrix (ECM), primarily consisting of type II collagen and proteoglycans such as aggrecan, which together provide tensile strength and resistance to compressive loading [11] (Figure 1).

Chondrocytes are the sole resident cell type and play a central role in maintaining cartilage homeostasis by regulating the balance between ECM synthesis and degradation. Under physiological conditions, this balance preserves the structural integrity and biomechanical function of the joint. Disruption of this homeostasis is a key initiating factor in osteoarthritis. Decreased anabolic activity and/or increased production of catabolic enzymes, including matrix metalloproteinases (MMPs) and a disintegrin-like and metalloproteinase with thrombospondin motif (ADAMTS), lead to progressive cartilage degradation [12]. Importantly, OA is now recognized as a whole-joint disease involving not only cartilage degeneration but also synovial inflammation and subchondral bone remodeling, which together contribute to joint dysfunction and disease progression [13]. These structural and cellular features of normal joints provide the essential foundation for understanding OA pathogenesis and for developing targeted therapeutic strategies, including gene therapy approaches aimed at restoring cartilage homeostasis.

## 3. Viral Delivery Systems

Viral gene therapy uses engineered viruses as vectors to deliver therapeutic genetic material into cells to modify disease-relevant biological processes. The commonly used viruses in gene therapy studies are AAV, lentivirus, and Ad [14]. These modified vectors transduce target cells to introduce a new or modified gene, replace a disease-causing gene with a healthy one, or silence a malfunctioning gene. AAV and lentiviral vectors can provide sustained transgene expression, but efficient cellular transduction does not ensure unhindered penetration through dense cartilage ECM [15,16].

### 3.1. Adeno-Associated Virus (AAV)

Due to its minimal pathogenicity and ability to establish long-term gene expression in different tissues, AAV has emerged as a pivotal delivery tool in clinical gene therapy for treating various diseases [10]. Several AAV-based medicines have received US FDA approval, and additional applications are under clinical investigation [5].

AAV is a small, non-enveloped virus of the Parvoviridae family, approximately 25 nm in diameter, with a T = 1 icosahedral capsid. Its genome consists of ~4.7 kb single-stranded DNA (ssDNA) flanked by inverted terminal repeats (ITRs), which are essential for viral genome replication, packaging, and persistence. Recombinant AAV (rAAV) vectors are generated by replacing the viral coding sequences (rep and cap genes) with a therapeutic transgene cassette while retaining the ITRs as the only cis-acting elements required for vector production and genome maintenance. Following cellular entry, the rAAV genome is transported to the nucleus, where it is converted into double-stranded DNA (dsDNA) and predominantly persists as episomal concatemers, enabling sustained expression without efficient integration into the host genome [17]. The packaging capacity of rAAV is limited to approximately 4.7 kb for ssAAV vectors, while scAAV vectors allow faster transgene expression at the expense of reduced cargo capacity (~2.4 kb) [18]. In addition, AAV capsids can be engineered to alter tropism, enhance transduction efficiency, and overcome biological barriers, making rAAV a versatile platform for in vivo gene therapies [19].

**Figure 2:**
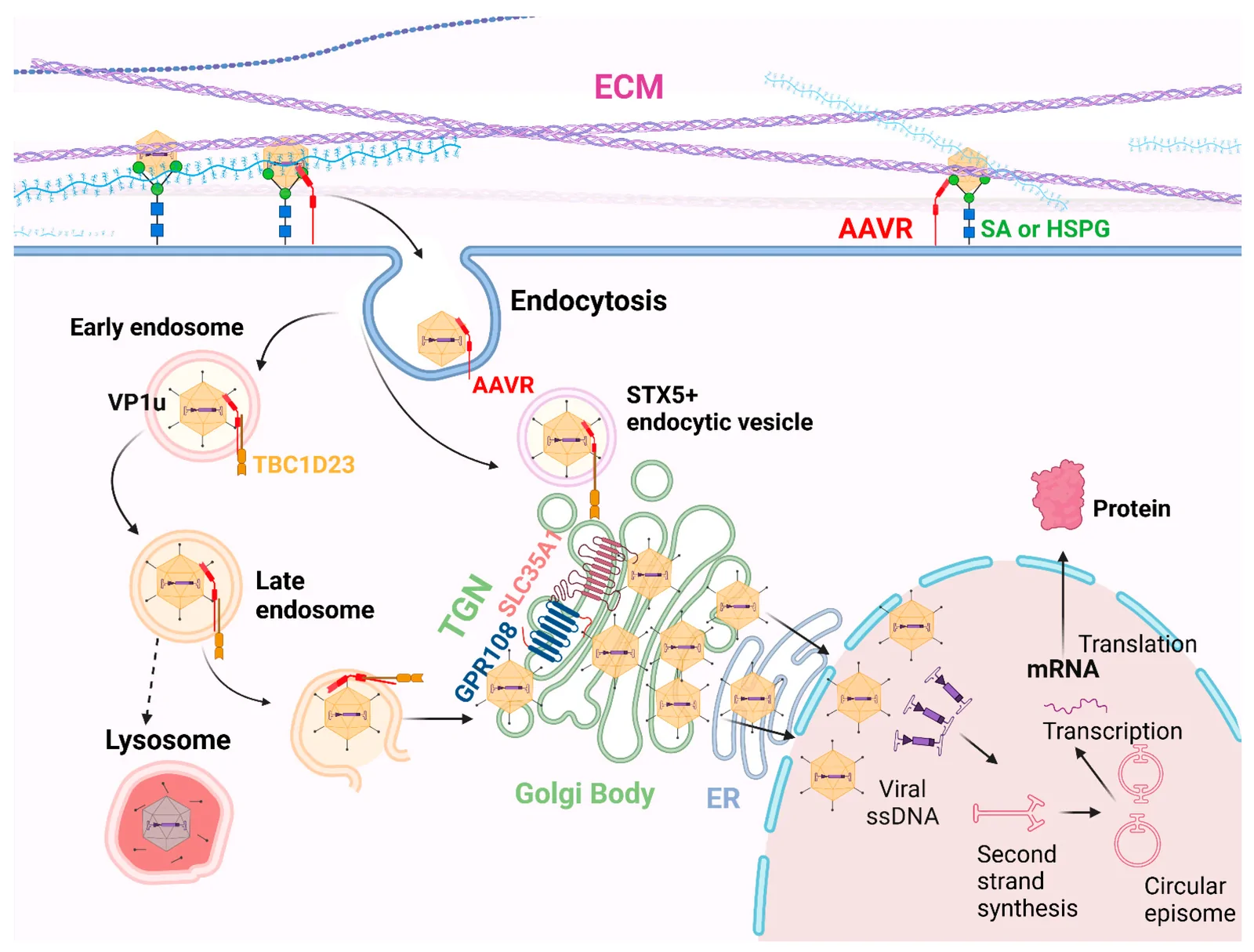
Proposed model of AAVR-mediated entry and intracellular trafficking of AAVR-dependent AAVs in chondrocytes. Schematic of AAV-mediated gene delivery to chondrocytes following intra-articular injection. AAV particles diffuse through the cartilage extracellular matrix (ECM), where the dense collagen II–proteoglycan network limits penetration to cells in superficial and intermediate zones.

As shown in Figure 2, at the cell surface, AAV binds to primary attachment factors such as sialic acids (SA) or heparan sulfate proteoglycans (HSPG) and engages entry receptors, such as AAV receptor (AAVR/KIAA0319L) [20,21], which facilitate internalization via the clathrin-independent carrier/glycosylphosphatidylinositol-enriched endocytic compartment (CLIC/GEEC) pathway [22]. After endocytosis, AAV traffics through Syntaxin 5-positive (STX5^+^) endocytic vesicles [23], or early and late endosomal compartments, where endosomal acidification promotes conformational changes in the capsid and exposure of the VP1 phospholipase A2 (PLA_2_) domain [24]. A subset of particles undergoes retrograde transport from endosomes to the *trans*-Golgi network (TGN), a pathway regulated by the AAVR and TBC1D23 interaction [25]. At the TGN, AAV particles interact with GPR108 and possibly SLC35A1 [26,27], and further undergo post-TGN trafficking through endoplasmic reticulum (ER) and are transported to the perinuclear region and enter the nucleus, where uncoating releases the ssDNA genome. The viral genome is converted to dsDNA and persists predominantly as episomal concatemers [28], enabling sustained transgene expression in chondrocytes. This proposed pathway draws largely on studies in other cell systems; its individual steps and relevance across AAV serotypes require validation in chondrocytes.

The use of AAV as a transgene delivery system has been studied for decades in rodent and large animal OA models [6,11]. AAV vectors exhibit broad tissue tropism, which can be further optimized through capsid serotype selection or engineering to enhance targeting efficiency. In the context of OA, intra-articular delivery of AAV allows localized gene transfer to synovial cells, and to a lesser extent, articular chondrocytes embedded within the dense ECM. However, the avascular nature and dense collagen–proteoglycan network of articular cartilage present a physical barrier to vector penetration, which remains a major limitation [29].

Preclinical studies have demonstrated that AAV-mediated delivery of therapeutic genes, particularly anti-inflammatory cytokines and anabolic factors, can reduce cartilage degeneration and joint inflammation in OA models [6,11]. Among these, IL-1Ra has been extensively investigated due to the central role of IL-1 signaling in OA pathogenesis. A recent open-label phase 1 trial demonstrated sustained AAV-mediated IL-1Ra expression in human OA knee joints, with no vector-related serious adverse events; improvements from baseline in pain and function were exploratory [10].

Despite these promising findings, several challenges remain for AAV-based OA gene therapy. Pre-existing neutralizing antibodies against AAV capsids may limit transduction efficiency and hinder repeat dosing, which is particularly relevant for chronic diseases such as OA [30,31]. In addition, improving vector penetration into cartilage and achieving efficient transduction of chondrocytes are critical barriers that are currently being addressed through capsid engineering and advanced delivery strategies.

### 3.2. Adenoviral Vectors

Adenoviral vectors (AdVs) are non-enveloped, dsDNA virus-based delivery systems that enable efficient gene transfer and high-level transgene expression in both dividing and non-dividing cells including mature chondrocytes. Expression duration depends on vector design and host immunity; first-generation vectors generally provide transient expression [32]. AdV-mediated nuclear factor-κBp65 (NF-κBp65)-specific siRNA (Ad-siRNANF-κBp65) has been shown to suppress the progression of early OA in a rat model [33]. The use of AdV to deliver basic fibroblast growth factor (bFGF), bFGF combined with IL-1Ra, and/or insulin-like growth factor-1 (IGF-1) in both human OA chondrocytes and rabbit OA models, promoted chondrocyte proliferation and ECM synthesis [34]. Adenoviral immunogenicity can trigger inflammation and limit repeat dosing [35]; high-capacity vectors differ from first-generation vectors and should be assessed separately.

### 3.3. Lentiviral Vectors

Lentiviral vectors are derived from HIV with a relatively large cargo capacity to integrate large transgenic fragments into the host genome, allowing long-term expression of therapeutic genes. Lentiviral vector-mediated Sox9 overexpression in IL-1β-treated mouse chondrocytes greatly improved cell survival through suppressing apoptotic signaling pathways. The beneficial effects of Sox9 overexpression were associated with up-regulated expression of type II collagen and aggrecan in the IL-1β-induced degenerated chondrocytes while suppressing inflammatory and pro-apoptotic molecules. Therefore, overexpression of Sox9 by lentiviral vector-mediated gene transfer is presented as a potential therapeutic approach [36]. Genomic integration raises concerns about insertional mutagenesis [37]. Insertion-associated hematologic malignancies have occurred after Lenti-D-transduced hematopoietic stem-cell therapy for cerebral adrenoleukodystrophy [38]. That experience concerns a different vector design and cell population and does not quantify risk for intra-articular lentiviral delivery; long-term OA-specific safety remains insufficiently characterized.

### 3.4. Retroviral Vectors

Retroviral vectors integrate their transgenes into the host-cell genome and can therefore provide stable gene expression. Conventional gammaretroviral vectors preferentially transduce dividing cells and have consequently been used mainly for ex vivo gene delivery to joints [30]. In a canine OA model, autologous synovial fibroblasts transduced with the MFG vector encoding human IL-1Ra reduced the severity of macroscopic and histologic cartilage lesions [39]. TissueGene-C (TG-C) combines unmodified allogeneic chondrocytes with irradiated, genetically modified GP2-293 cells expressing TGF-β1 [40]; this cell-mediated approach has advanced through clinical trials [41]. However, cell expansion and processing requirements, manufacturing complexity, and integration-associated safety concerns limit the broader application of retroviral vectors for OA.

## 4. Non-Viral Delivery Systems

Non-viral gene delivery systems use physical methods or chemical carriers, including lipid and polymer nanoparticles, to transport nucleic acids into cells. They offer flexible payload design and do not require a viral capsid, but delivery efficiency, expression duration, and inflammatory or cytotoxic effects depend on the formulation and cargo.

### 4.1. Physical Methods

Physical methods, including electroporation, gene gun, magnetofection, ultrasound, microinjection, etc., have been used for gene transfer, mainly ex vivo in cultured cells to create temporary membrane pores for uptake of genetic molecules. Even though these methods have advantages of flexible cargo capacity and avoidance of viral-vector immunity, their major drawbacks are cell damage, limited control of the amount and efficiency of the transferred gene(s), especially for the cells embedded deeply in host tissues, which limits their clinical use [42].

### 4.2. Chemical Carriers

Chemical carriers are engineered particles, mainly nanoparticles based on lipids, polymers, or peptides, that are able to carry and deliver nucleic acid into the cells. Among them, lipid nanoparticles (LNPs) are advanced vehicles that can be formulated to deliver DNA, mRNA, siRNA, or gene-editing components, which renders the use of LNPs not only for mRNA vaccines but also for gene therapy [43]. For example, repeated intra-articular *Fgf18* mRNA-LNP administration reduced cartilage degeneration and synovitis in mouse surgical and aging-associated OA models [44]. These are preclinical findings. Joint retention, cartilage access, cellular uptake, and intracellular cargo release must each be optimized; efficient delivery in mice does not establish efficacy in human OA.

### 4.3. Extracellular Vesicles (EVs)

EVs are membrane-bound nanoscale particles released by cells that carry proteins, lipids, and nucleic acids from the parent cell. Exosomes are a major subtype of EVs. EVs contain a diverse range of molecules derived from their parent cells and function as carriers of bioactive cargos, delivering them to target cells locally or at distant sites through circulation, thereby mediating intercellular communication [45]. Engineered EVs can carry nucleic acids, although reproducible production, cargo loading, and tissue targeting remain challenges. Charge-reversed exosomes delivered reporter mRNA and supported eGFP expression in mouse cartilage [29]. A separate study delivered IL-1Ra protein and reduced cytokine-induced cartilage catabolism in bovine explants [46]; this supports protein delivery rather than gene transfer.

### 4.4. Biomaterial-Assisted Delivery

Biomaterial-assisted delivery represents an emerging strategy for the controlled local release of small molecule drugs, cell delivery and gene therapy in OA treatments. As secondary delivery matrices, biomaterials can function as localized depots that prolong intra-articular retention, protect gene cargos from degradation, and regulate their spatial and temporal release. Injectable hydrogels based on hyaluronic acid, alginate, gelatin, chitosan, polyethylene glycol, and other natural or synthetic polymers are useful because they can be administered minimally invasively and engineered with tunable degradation, mechanical adaptability, and stimulus-responsive properties [47]. In gene therapy applications, hydrogels can be used to encapsulate nucleic acids or as secondary delivery platforms for lipid or polymeric nanoparticles, or extracellular vesicles, thereby combining efficient intracellular delivery with prolonged joint retention. For example, MMP-responsive silk-fibroin hydrogels containing LNPs loaded with circular RNA encoding the IκBα super-repressor inhibited NF-κB signaling and reduced cartilage damage in an OA rat model, with additional evaluation in human OA cartilage ex vivo [48]. Lentivirally engineered bone marrow mesenchymal stem cells seeded onto hydrogel microspheres have also been used for sustained EV-mediated miRNA delivery to cartilage in a rat OA model; this is a hybrid ex vivo gene-modified cell and EV delivery strategy [49]. Biomaterials can also be integrated with tissue-engineering scaffolds to provide spatially controlled gene expression and promote cartilage regeneration, particularly for focal cartilage defects [50]. Thus, hydrogel- or scaffold-assisted delivery can address several limitations of intra-articular non-viral gene therapy—including rapid clearance, limited cargo stability, and insufficient local exposure—while providing an adaptable platform for combining gene modulation with tissue regeneration. Nevertheless, challenges remain in achieving reproducible release kinetics, efficient penetration of cartilage extracellular matrix, intracellular delivery and endosomal escape, and scalable manufacturing, which will be critical for clinical translation. These approaches remain preclinical.

The major differences between viral vectors and non-viral carriers are summarized in Table 1.

**Table 1:** Comparative overview of viral and non-viral gene-delivery approaches for OA.

Feature	Viral Vectors	Non-Viral Carriers
Delivery efficiency	Varies with capsid or envelope, target cell, and tissue.	Varies with formulation, cargo, uptake, and intracellular release.
Expression duration	AAV: often prolonged; AdV: transient; lentiviral and ꝩ-retroviral vectors: persistent.	mRNA/siRNA effects: usually transient; plasmid expression: variable. Repeat dosing may be needed.
Immunogenicity	AAV capsid immunity can limit redosing, and AdV-induced inflammation depends on design.	Carrier components and nucleic acids can cause innate immune activation or toxicity.
Integration and safety	AAV/AdV: episomal; lentiviral/ꝩ-retroviral vectors: integrating, with insertional risk.	Plasmid, mRNA, and siRNA delivery does not require integration; editing cargo can cause durable DNA changes.
Cargo capacity	AAV: ~4.7 kb; scAAV: ~2.4 kb; regular AdV: ~8 kb, gutless AdV: ~36 kb; lentiviral/ꝩ-etroviral: ~8 kb.	Formulation-dependent; larger cargo can impair loading, stability, and delivery.
Cartilage access	Limited by dense ECM; varies with particle properties, cartilage integrity and zone, dose, and formulation.	Limited by dense ECM; varies with particle size, charge, targeting, and retention.

## 5. Delivery Methods for OA Therapy

**Figure 3:**
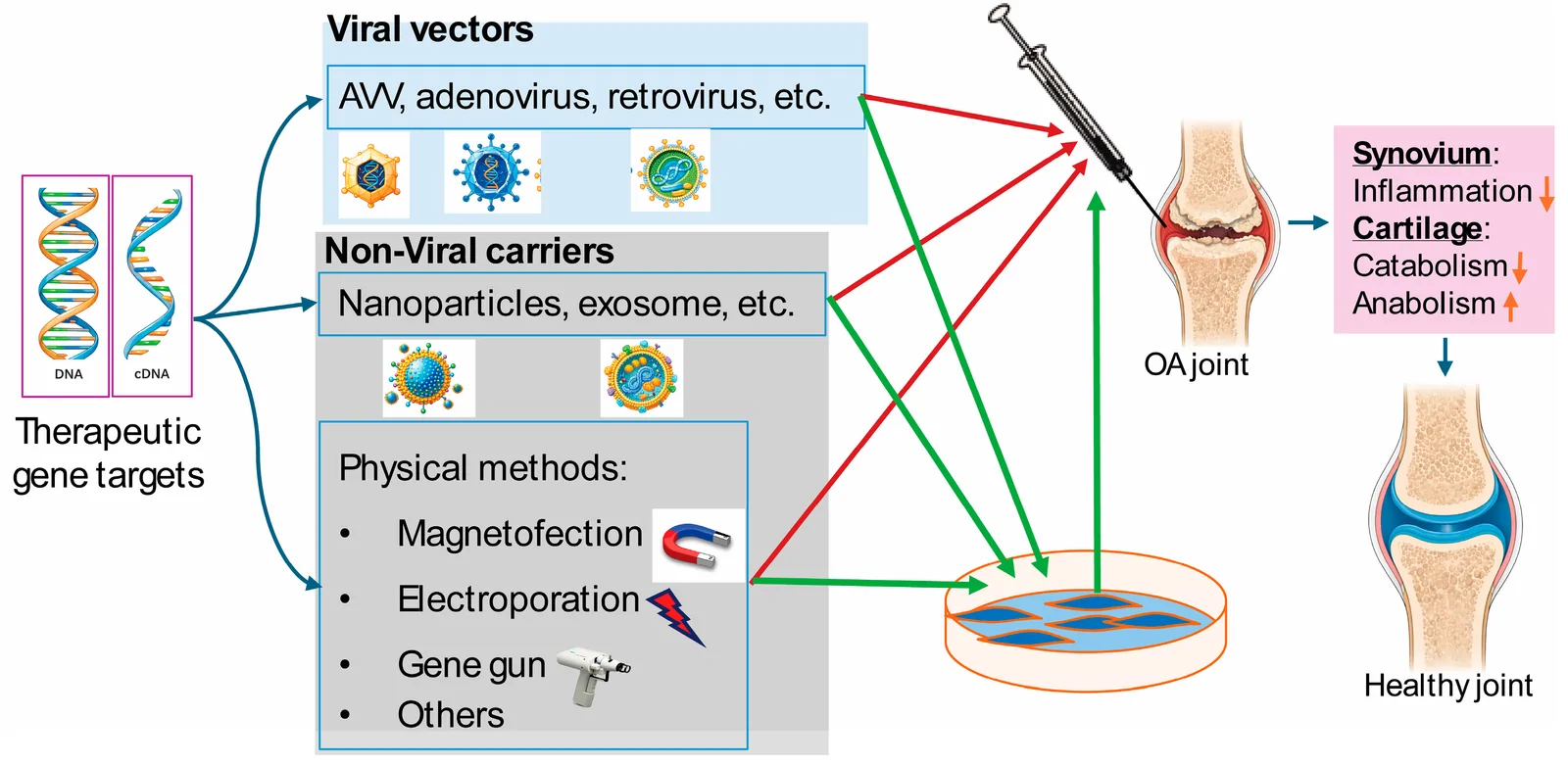
Schematic overview of gene delivery strategies targeting synovium and cartilage using DNA or mRNA payloads for OA treatment. These approaches can be applied either directly in vivo (red solid lines) via intra-articular injection or ex vivo (green dotted lines) through modification of cells followed by transplantation. Therapeutic gene delivery aims to reduce synovial inflammation and restore cartilage homeostasis, characterized by decreased catabolism and increased anabolism, thereby promoting articular tissue repair toward a healthy joint.

There are two approaches for transferring the transgene(s) or proteins into OA joints. One is to deliver the therapeutic material directly into the joint cavity, usually through intra-articular injection. This method is convenient, relatively cheap and less time-consuming. The other is an ex vivo-mediated method which first expands the cells in vitro, then the therapeutic materials are introduced into the cells, and finally the targeted cells are injected into the joint. The ex vivo method is technically cell therapy with engineered gene expression. The general vector delivery strategies are summarized in Figure 3.

Although the ex vivo cell expansion step can significantly increase the number of cells, it also increases the cost and the risk of exposure to environmental pathogens. Moreover, even though it is possible to combine the use of physical methods and the advantage of using stem cells for specific cell differentiation, no control has been developed on the fate of the injected cells, such as survival, integration, differentiation, and dedifferentiation of transplanted stem cells.

## 6. Therapeutic Targets for OA Therapy

Although OA has long been recognized as a “wear and tear” mechanical disease of the joint, biological studies of OA in human and animal models have revealed an increasing number of molecules (genes) that are involved in OA pathogenesis as summarized in Table 2. These findings identify candidate pathways for intervention, singly or in combination. Expression associations and genetic perturbation studies do not by themselves establish an effective or safe therapeutic target; results from animal models require validation in human OA.

**Table 2:** Selected OA-associated targets and supporting biological evidence.

Gene (Protein/Alias)	Tissue or Experimental System	Species	Evidence Relevant to OA	References
*DKK1*	Plasma and synovial fluid	Human	DKK1 protein levels were inversely correlated with radiographic severity of knee OA.	[51]
*EGR1*	Knee articular cartilage	Human	Increased EGR1 expression was observed in OA cartilage.	[52]
*FOS* (c-Fos)	Knee articular cartilage	Human	The proportion of FOS-positive chondrocytes increased with OA severity.	[53]
*FOSL2*	Knee articular cartilage	Human	FOSL2 mRNA was downregulated in an OA cartilage RNA-sequencing cohort.	[54]
*GDF5*	Genetic association and chondrogenic-cell reporter assays	Human	The OA-associated rs143383 susceptibility allele showed reduced promoter activity in chondrogenic cells.	[55]
*HDAC4*	Articular cartilage/chondrocytes	Human	Reduced HDAC4 in OA cartilage was associated with increased RUNX2 and catabolic markers; HDAC4 overexpression suppressed these markers in cultured OA chondrocytes.	[56]
*EPAS1* (HIF-2α)	Articular cartilage/chondrocytes	Human	HIF-2α expression was increased in human OA cartilage.	[57]
*IL10*	Articular cartilage/chondrocytes	Human	IL-10 mRNA was higher in OA chondrocytes; intracellular IL-10 protein was highest in the most damaged OA regions.	[58]
*IL1RN* (IL-1Ra)	Genomic DNA; synovial fluid	Human	IL1RN variants were associated with radiographic knee OA severity; a protective haplotype was associated with greater joint-space width and lower synovial-fluid inflammatory cytokines.	[59]
*JUN*	Knee synovial tissue	Human	JUN expression was elevated in OA synovial tissue.	[60,61]
*JUND*	Knee articular cartilage	Human	JUND mRNA was decreased in OA cartilage, including validation by qRT-PCR.	[62]
*KLF4*	Cultured primary chondrocytes treated with IL-1β	Human	Lentiviral KLF4 overexpression reduced apoptosis and mitochondrial injury in an in vitro OA model.	[63]
*MYC*	Knee synovial tissue	Human	MYC expression was elevated in OA synovial tissue.	[61]
*PRG4* (lubricin)	Synovial fluid	Human	Some OA synovial-fluid samples had reduced PRG4 protein and impaired cartilage boundary lubrication, which improved with PRG4 supplementation.	[64]
*RELA* (NF-κB p65)	Knee articular cartilage	Human	RELA mRNA was downregulated in an OA cartilage RNA-sequencing cohort; this does not directly measure NF-κB activation.	[54]
*RUNX2*	Articular cartilage/chondrocytes	Human	RUNX2 expression was increased in OA cartilage together with MMP13 and hypertrophic markers.	[56]
*SOX9*	Knee articular cartilage	Human	SOX9 mRNA was reduced in OA cartilage compared with age-matched control cartilage.	[65]
*TGFB1*	Articular cartilage and synovium	Human	The OA risk variant rs75621460 altered enhancer activity; genetic and epigenetic effects on TGFB1 regulation differed between joint tissues.	[66]
*THBS1* (TSP-1)	Articular cartilage	Human	TSP-1 expression/staining increased in mild-to-moderate OA but decreased in severe OA cartilage.	[67]
*Ctnnb1* (β-catenin)	Articular chondrocytes/cartilage	Mouse	Conditional β-catenin activation caused an OA-like phenotype; transgenic inhibition of β-catenin signaling increased chondrocyte apoptosis and cartilage destruction.	[68,69]
*Epas1* (HIF-2α)	Articular cartilage/chondrocytes	Mouse	Experimental HIF-2α overexpression promoted cartilage destruction; genetic reduction of Epas1 suppressed experimental OA.	[57]
*Nfatc2* (NFAT1)	Adult articular chondrocytes; hip and knee cartilage	Mouse	Nfatc2 deficiency caused spontaneous OA-like cartilage degradation and associated joint changes.	[70]
*Nkx3-2* (BAPX1)	Knee articular cartilage	Mouse	NKX3-2-positive articular chondrocytes were reduced in experimental OA.	[71]
*Runx2*	Articular cartilage/chondrocytes	Mouse	RUNX2 was induced after joint instability; heterozygous Runx2 deficiency reduced cartilage destruction and osteophyte formation.	[72]

Note: These are selected examples, not a list of clinically validated targets. The evidence column distinguishes expression or protein-level associations, genetic associations, and experimental perturbations. In the first column, human gene symbols are uppercase and italicized; mouse gene symbols use initial-capital italics. Protein names and common aliases are shown in roman type.

## 7. Preclinical and Clinical Studies of Gene Therapy for OA

Preclinical studies have investigated OA-related targets in animal models and ex vivo joint tissues or cells; these include target-biology experiments as well as therapeutic delivery studies. These targets and pathways can be grouped into three categories: (1) Anabolic or anti-inflammatory factors such as PRG4, SOX9, and NFAT1 [64,70,73,74,75,76]; (2) Catabolic pathways involving IL-1 signaling, RUNX2, and HIF-2α [34,57,72]; and (3) Context-dependent pathways such as β-catenin signaling, for which both conditional activation and transgenic inhibition in mouse chondrocytes caused cartilage degeneration [68,69]. Table 3 summarizes selected studies delivering nucleic acids or gene products to joints in animal models of OA or joint injury, spanning multiple platforms and species. NFAT1, RUNX2, and β-catenin are omitted because these target genes were cited from genetically modified animal models rather than intra-articular delivery studies. The table is not exhaustive and does not compare efficacy across platforms.

**Table 3:** Selected preclinical gene and nucleic-acid delivery studies in OA animal models.

Animal Model	Therapeutic Cargo or Target	Delivery Platform	Main Preclinical Finding	Reference
Dogs; experimental OA	IL1RN encoding IL-1Ra	MFG retrovirus; ex vivo-modified synovial fibroblasts	Reduced macroscopic and histologic cartilage lesion severity compared with controls.	[39]
Rats; surgical OA	IL1RN encoding IL-1Ra (FX201)	High-capacity adenovirus	Reduced cartilage, bone, and synovial changes at week 12 after surgery; limited peripheral vector dissemination.	[77]
Horses; osteochondral fragment model	IL-1Ra expression cassette	Self-complementary AAV	Improved lameness, reduced prostaglandin E2, and improved cartilage and subchondral-bone histology.	[78]
Rats; ligament transection and partial meniscectomy	NF-κB p65 silencing	Adenoviral vector expressing p65-targeting siRNA	Reduced early cartilage degradation, synovial inflammation, and synovial-fluid IL-1β/TNF-α.	[33]
Mice; DMM and aging-associated OA	FGF18 mRNA	Intra-articular LNPs; weekly dosing	Reduced cartilage degeneration and synovitis; improved gait and reduced pain hypersensitivity in the DMM model.	[44]
Rabbits; anterior cruciate ligament transection	PRG4 encoding lubricin	AAV; intra-articular	Increased lubricin expression and protected cartilage from degeneration when given immediately after surgery; efficacy was lost when treatment was delayed by 2 weeks	[79]
Mice; two surgical post-traumatic OA models	PRG4 and IL1RN encoding IL-1Ra, delivered together	Helper-dependent adenovirus; intra-articular	Combined delivery preserved articular cartilage better than either monotherapy and prolonged protection against hyperalgesia	[80]
Sheep; focal osteochondral defect with perifocal OA assessment	SOX9	rAAV; applied to the defect	Improved cartilage repair scores and reduced perifocal osteoarthritic changes at 6 months versus rAAV-lacZ control	[81]
Mice; surgical OA	HIF-2α silencing	Chondrocyte-homing nanoparticles carrying siRNA; intra-articular	Reduced catabolic mediators, cartilage degeneration and synovial inflammation	[82]

Table 4 summarizes eight selected registered knee-OA clinical trials, including a withdrawn study. Registry records were last checked on 28 September 2026; the list is not exhaustive.

TG-C delivers TGF-β1 through genetically modified cells and has been evaluated in phase III trials, while AAV-based anti-inflammatory therapies are in early-phase studies. Randomized TG-C data support symptomatic improvement, but reported MRI structural differences were not statistically significant [41]. For PCRX-201, published phase 1 results describe local effusions and exploratory symptom improvements through 104 weeks; the absence of a placebo group and substantial attrition limit efficacy interpretation [83]. Sustained transgene expression or reduced pain should not be equated with demonstrated structural disease modification.

**Table 4:** Selected registered gene-therapy clinical trials for knee OA.

Trial/Source	Platform/Target	Phase; n; OA Grade; Status	Endpoints and Follow-Up	Safety Findings	Efficacy Findings
NCT02072070 [41]	Ex vivo retroviral cell product; TGF-β1 (TG-C)	III; 163 actual; KL3; completed	Primary: IKDC/VAS at 52 weeks. Structural: MRI/joint-space width (secondary). Follow-up: 52 weeks.	Edema, arthralgia, swelling and injection-site pain reported.	Published: pain/function improved versus placebo; structural trends were not statistically significant.
NCT02790723 [10,84]	scAAV2.5; IL1RN/IL-1Ra	I; 9 actual; KL2–3; completed	Primary: safety. Structural: exploratory X-ray/MRI. Published follow-up: 52 weeks.	No related serious AEs; two possibly related effusions resolved; neutralizing antibodies increased.	Published: sustained synovial IL-1Ra and improved symptoms from baseline; uncontrolled study.
NCT04124042 [85]	Plasmid DNA; IL-10 variant (XT-150)	II; 289 actual; KL2–3; completed	Primary: WOMAC pain at day 180 and AEs. Structural: none registered. Follow-up: 360 days.	Registry results: no treatment-related serious AEs or anti-IL-10 antibodies.	Registry results: neither dose significantly improved the primary day-180 pain outcomes versus placebo.
NCT04119687 [83,86]	High-capacity Ad5; IL1RN/IL-1Ra (PCRX-201, formerly FX201)	I; 72 actual; KL2–4; active, not recruiting	Primary: AEs through 104 weeks. Structural: exploratory radiography/MRI. Follow-up: 5 years planned.	No treatment-related serious AEs; effusions common (22/36 without and 13/36 with steroid pretreatment).	Published: symptom improvements in remaining participants; uncontrolled, with 45.8% discontinuation by week 104. Structural benefit unproven.
NCT07198555 [87]	AAV; anti-inflammatory transgene (BBM-A101; not specified in registry)	I; 81 planned; KL2–3; recruiting	Primary: toxicity at 4 weeks; AEs at 52 weeks. Structural: joint-space width (secondary). Follow-up: 5 years.	Results not yet available for this trial.	Results not yet available for this trial.
NCT05835895 [88]	scAAV2.5; IL1RN/IL-1Ra (GNSC-001)	Ib; 67 actual; KL2–3; active, not recruiting	Primary: AEs at 12 months. Structural: none registered. Safety follow-up: 48 months planned.	Conference report: no related serious AEs; mostly mild/moderate knee AEs.	Conference report: sustained synovial IL-1Ra; exploratory symptom improvements. Confirmatory efficacy results unavailable.
NCT05454566 [89]	AAV5.2; truncated NKX3-2/BAPX1 (ICM-203)	I/II; 0 actual; planned KL2–3; withdrawn	Planned primary: AEs, pain/function, analgesic use, MRI and joint-space width through 52 weeks.	Not assessed: withdrawn before enrollment.	No results: sponsor did not proceed with this US trial.
NCT03383471 [90]	Ex vivo retroviral cell product; TGF-β1 (Invossa K)	III; 146 planned; KL2; unknown status	Primary: IKDC/VAS at 52 weeks. Structural: MRI/joint-space width (secondary). Follow-up: 52 weeks planned.	Results not yet available for this trial.	Results not yet available for this trial.

Registry records checked on 28 September 2026. Eight selected knee-OA trials are shown, not an exhaustive census. Enrollment is actual unless marked planned. KL, Kellgren–Lawrence grade; AE, adverse event; IKDC, International Knee Documentation Committee; VAS, visual analog scale; WOMAC, Western Ontario and McMaster Universities Osteoarthritis Index. Clinical safety/pain endpoints are distinguished from structural assessments. NCT02790723 specifies 53 weeks in the registry; published follow-up was 52 weeks. Unknown status denotes an outdated registry record, not confirmed completion.

Earlier IL-1Ra gene transfer in two patients with rheumatoid arthritis [91] is not included as OA efficacy evidence. ART-I02 (AAV5-mediated IFN-β expression under an NF-κB-responsive promoter; NCT02727764 [92]) had eligibility including rheumatoid arthritis or OA of the hand. Enrollment stopped after four participants because persistent local adverse events occurred in three [93]; these mixed hand-arthritis data are considered separately from knee-OA trials.

For Table 4, GNSC-001 phase 1b findings are from Keravala et al., ACR Convergence 2025, abstract 0310, Arthritis Rheumatol. 2025; 77 (suppl 9), and remain conference-level evidence. The ICM-203 target is described by Heald et al., Osteoarthritis Imaging. 2025; 5 (suppl 1):100291; that abstract reports a separate trial, NCT04875754, whose outcomes are not attributed to the withdrawn US trial.

## 8. Barriers to Clinical Translation and Future Directions

Despite encouraging results, several challenges remain for OA gene therapy:

- Immune responses to viral vectors: Many patients have pre-existing neutralizing antibodies to common AAV capsids. Clinical trials in 2025 (e.g., sc-rAAV2.5IL-1Ra) have confirmed that even with intra-articular injection, patients develop systemic and local neutralizing antibodies [10]. This makes repeat dosing, often necessary for chronic OA, a major regulatory and immunological hurdle.
- Limited penetration in dense cartilage tissue: The dense collagen–proteoglycan matrix limits access to chondrocytes. Capsid engineering and carrier modification may improve delivery, but penetration depends on cartilage integrity, depth, particle size, charge, and formulation [19,29,44].
- Dosing optimization for efficacy and safety, especially for the long-term monitoring considerations.
- Biomarker development and advanced imaging for efficacy assessment.
- Manufacturing costs: Clinical-grade viral vector production remains prohibitively expensive for OA gene therapy.
- Patient heterogeneity: OA is no longer viewed as a single disease but a collection of "phenotypes" (e.g., inflammatory, metabolic, post-traumatic). A single gene (like IL-1Ra) may not work for all phenotypes. This has led to a move toward combinatorial gene therapy (e.g., delivering both anti-inflammatory and anabolic genes simultaneously) or target upstream transcription factors which regulate both downstream anabolic and catabolic genes.

To overcome these barriers, the future directions should include:

- Evaluating biomaterial-assisted delivery to improve local retention and controlled release: benefits for penetration and immune responses require direct testing for each formulation.
- Combining viral vectors with EVs: EV-associated AAV has improved delivery or immune evasion in non-OA preclinical systems [94,95,96,97,98]. These findings motivate investigation in joint tissues, but enhanced cartilage penetration, efficacy, and safety after intra-articular administration remain to be demonstrated. EV association should not be assumed to confer the same benefit across tissues or formulations.
- Personalized therapy: It is important to subgroup the OA patients based on specific molecular risk factors for the selection of gene target(s), which will help to optimize the outcomes of clinical trials [99].
- Introducing gene editing for OA therapy: Genome editing can modify endogenous DNA rather than supply an additional expression cassette [100]. OA is generally multifactorial, so target selection, delivery to the relevant joint cells, off-target effects, and long-term safety require evaluation before clinical application.

## 9. Conclusions

Gene therapy is an investigational approach with disease-modifying potential for OA. Viral vectors, particularly AAV, can support sustained local transgene expression, while non-viral platforms offer flexible nucleic-acid delivery. Cartilage access and safety depend on the vector or carrier, cargo, and tissue context. Preclinical gene therapy studies have confirmed delivery efficiency and anti-OA biological activity while clinical trials demonstrated feasibility and symptomatic benefits. However, durable structural disease modification has not been well established. Important translational barriers include immune responses, limited cartilage penetration, dosing, manufacturing, and patient heterogeneity. Further controlled trials with structural endpoints and long-term follow-up are needed to determine the clinical value of these gene therapy approaches.

## Data Availability

No new data were created or analyzed in this study. Data sharing is not applicable to this review article.
